# Chlorhexidine bathing of the exposed circuits in extracorporeal membrane oxygenation: an uncontrolled before-and-after study

**DOI:** 10.1186/s13054-020-03310-w

**Published:** 2020-10-06

**Authors:** Hye Ju Yeo, Dohyung Kim, Mihyang Ha, Hyung Gon Je, Jeong Soo Kim, Woo Hyun Cho

**Affiliations:** 1 Department of Pulmonology and Critical Care Medicine, Research Institute for Convergence of Biomedical Science and Technology, Pusan National University Yangsan Hospital, Geumo-ro 20, Beomeo-ri, Mulgeum-eup, Yangsan-si, Gyeongsangnam-do 626-770 Republic of Korea; 2 Department of Thoracic and Cardiovascular Surgery, Pusan National University Yangsan Hospital, Yangsan-si, South Korea; 3 Interdisciplinary program of Genomic Science, Pusan National University, Yangsan-si, South Korea; 4 Division of cardiology, Department of Internal Medicine, Pusan National University Yangsan Hospital, Yangsan-si, South Korea

**Keywords:** Chlorhexidine disinfection, ECMO, Catheter, Infection, Mortality

## Abstract

**Background:**

Although the prevention of extracorporeal membrane oxygenation (ECMO) catheter-related infection is crucial, scientific evidence regarding best practices are still lacking.

**Methods:**

We conducted an uncontrolled before-and-after study to test whether the introduction of disinfection with 2% chlorhexidine gluconate (CHG) and 70% isopropyl alcohol (IPA) of the exposed circuits and hub in patients treated with ECMO would affect the rate of blood stream infection (BSI) and microbial colonization of the ECMO catheter. We compared the microbiological and clinical data before and after the intervention.

**Results:**

A total of 1740 ECMO catheter days in 192 patients were studied. These were divided into 855 ECMO catheter days in 96 patients before and 885 ECMO catheter days in 96 patients during the intervention. The rates of BSI were significantly decreased during the intervention period at 11.7/1000 ECMO catheter days before vs. 2.3/1000 ECMO catheter days during (difference 9.4, 95% confidence interval (CI) 1.5–17.3, *p* = 0.019). Furthermore, the colonization of the ECMO catheter was similarly significantly reduced during the intervention period at 10.5/1000 ECMO catheter days before vs. 2.3/1000 ECMO catheter days during intervention (difference 8.3, 95% CI 0.7–15.8, *p* = 0.032). Hospital mortality (41.7% vs. 24%, *p* = 0.009) and sepsis-related death (17.7% vs. 6.3%, *p* = 0.014) were also significantly decreased during intervention.

**Conclusion:**

Extensive disinfection of exposed ECMO circuits and hub with 2% CHG/IPA was associated with a reduction in both BSI and microbial colonization of ECMO catheters. A further randomized controlled study is required to verify these results.

**Trial registration:**

KCT 0004431

## Background

Extracorporeal membrane oxygenation (ECMO) is increasingly being used worldwide to save patients with severe cardiorespiratory failure. Depending on the patient’s condition, ECMO treatment may last several days or longer, but its presence is a major risk factor for blood stream infection (BSI). In previous studies, it was found that 19–32% of inserted ECMO catheters were colonized with potentially pathogenic bacteria at the point of removal and that about 10% were associated with ECMO device infection [1, 2]. While BSI occurrence during ECMO is a serious complication associated with significant morbidity and mortality, its prevalence has been reported to be as much as 3–18% in adults [3]. Removal of the catheter is the first approach in the treatment of any catheter-related infection; however, it is difficult to remove the presumably infected ECMO catheters if the patient is not ready for weaning off ECMO due to cardio-respiratory dysfunction. Currently, the routine use of antibiotic prophylaxis for patients on ECMO support is not recommended [4]. It is likely to only increase the risk of resistant strain development and can potentially lead to yeast overgrowth [4]. Given that the predominant organisms colonizing ECMO catheters were found to be gram-positive cocci and Candida species [1, 2], it seems likely that skin bacteria traverse the insertion site onto the catheter, where they colonize the circuit and act as a focus for BSI. Furthermore, manipulation of hub for the use of renal replacement therapy (RRT) during ECMO, or for oxygenator function test, and exchange of oxygenator or catheters potentially pose a risk of BSI.

Chlorhexidine bathing has been shown to be an effective measure in reducing levels of pathogens on the skin, and it can also prevent colonization and central line associated BSI [5–7]. In particular, 2% chlorhexidine gluconate (CHG)/isopropyl alcohol (IPA) was proven effective in reducing the viability of biofilms formed by skin microflora [8]. Considering that manipulation of the line and the hub of ECMO is inevitable and that BSI is a common problem during ECMO, it is necessary to expand the dressing area from the insertion sites to the port of entry and hub of ECMO. Adequate disinfection of the exposed circuits and hub may arguably be the most important component of ECMO catheter care to prevent catheter colonization and related BSI. In this study, we investigated whether disinfection with 2% CHG/IPA of all exposed circuits and hubs impacts on the rate of BSI and ECMO catheter colonization.

## Methods

### Study design

**Fig. 1:**
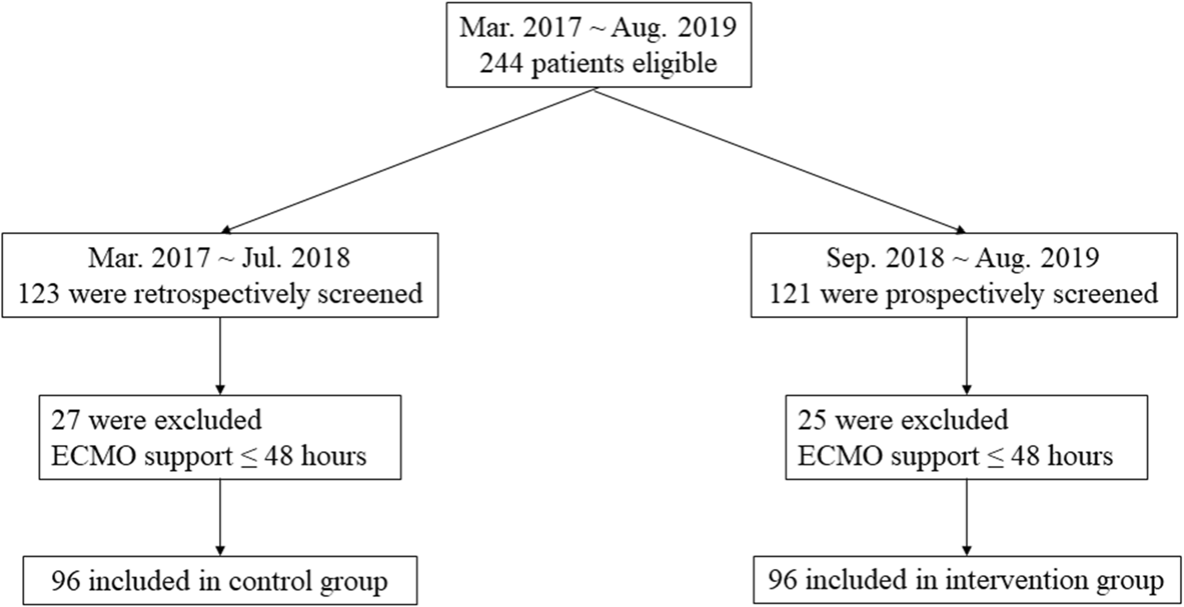
Patient enrollment diagram

This was an uncontrolled, single center, before-and-after study. Patients who required ECMO for cardiogenic or respiratory failure and were older than 18 years were screened for eligibility. Exclusion criteria included a termination of ECMO support within 48 h and presence of chlorhexidine allergy. The intervention was a daily disinfection of all exposed circuits and hub including ECMO catheter insertion sites with 2% CHG/IPA during ECMO support. An additional file shows this in more detail (see Additional file 1). In order to minimize the performance bias, we trained the intervention practice for one month in August 2018. The intervention group was prospectively enrolled after obtaining consent from the patient or guardian from September 2018 to August 2019 and collected data. The control group was retrospectively collected data on patients who performed ECMO before the intervention from March 2017 to July 2018. During the pre-intervention period, standard practice was for all ECMO patients to receive daily 2% CHG disinfection at the ECMO catheter insertion site according to the infection precaution policy of the hospital [4]. A total of 244 patients were screened during the study period (Fig. 1). Of those, 52 patients who conducted ECMO within 48 h were excluded, and 192 were enrolled in this study. Except for the intervention, all patient care and ECMO management were performed identically. Basically, all patients with central lines received daily 2% CHG bathing to clean neck and chest. The dialysis line was connected to the ECMO hub when RRT was required during ECMO, and post-membrane blood gas analysis was performed when oxygenator dysfunction was suspected. Also, oxygenator was exchanged when oxygenator dysfunction or massive clot was observed. There were no differences in general practice and diagnostic approach since reaching the learning curve for multidisciplinary team setup in ECMO [9]. This project was approved by the Pusan National University Yangsan Hospital Institutional Review Board (05-2018-149) and the study was registered on the Clinical Research Information Service (KCT 0004431). In the intervention group, written informed consent for enrollment or consent to continue and use patient data was obtained from each patient or their legal surrogate. The control group was waived from consent due to retrospective data collection by the IRB.

### Outcome measures

The primary outcome was the incidence of BSI during ECMO. Consistent with Centers for Disease Control and Prevention/National Healthcare Safety Network criteria, BSI during ECMO was defined as at least one of the following criteria, measured from 48 h after insertion of ECMO catheters to 1 day after termination of ECMO: (1) a recognized bacterial/ fungal pathogen cultured from one or more blood cultures that is not related to an infection at another site and (2) a common commensal organism (e.g., coagulase-negative staphylococcus) in two or more blood cultures collected on different days or from different sites that is not related to an infection at another site and that occurs in the setting of one of the following signs/symptoms: fever (> 38.0 °C), chills, or hypotension [10, 11]. Secondary outcomes were colonization of ECMO catheters, overall mortality, sepsis-related death, ECMO complications, and microbiology of catheter colonization. Catheter colonization was defined as a positive culture of ECMO catheters at the time of removal. Growth of > 15 colony forming units (CFU) from a 5-cm segment of the catheter tip by semiquantitative (roll-plate) culture or growth of > 10^2^ CFU from a catheter by quantitative (sonication) broth culture reflects catheter colonization [12]. Other central venous catheter-related infections were defined by the clinical practice guidelines of the Infectious Disease Society of America (IDSA) [12]. Ventilator-associated pneumonia (VAP) was diagnosed by the IDSA/ATS guidelines [13]. Sepsis-related death was defined as the immediate cause of death was sepsis, or sepsis was a factor in a chain of events leading to death.

### ECMO management

All ECMO catheter insertions were performed using a strict aseptic technique by standardized guidelines by an experienced cardiothoracic surgeon using a Seldinger approach under ultrasound guidance [14]. The optimal insertion site for each individual patient was selected by experienced cardiothoracic surgeons. All involved personnel were trained in catheter maintenance procedure, and standardized continuous catheter care was provided according to the Extracorporeal Life Support Organization guidelines [14]. The ECMO system consisted of a polymethylpentene fiber oxygenator system (QUADROX*PLS*; Maquet Inc., Hirrlingen, Germany) with simplified Bioline-coated circuits (Maquet Inc., Rastatt, Germany). All patients were supported with centrifugal pumps (Maquet Inc., Hirrlingen, Germany). Our hospital had no set protocol for antimicrobial prophylaxis in patients receiving ECMO. Standardized, empirical antibiotic therapy with broad-spectrum drugs was initiated according to anti-infection guidelines for suspected and confirmed infections, when deemed necessary by the attending intensivist. During ECMO support, antibiotics were adjusted according to the clinical course of the disease and culture results. All insertion sites were regularly checked for potential dressing contamination. If clinically indicated, the perfusionists and cardiothoracic surgeon changed the dressing, and cleaned the skin site additionally. There was effective removal of urine and feces.

### ECMO catheter collection and preparation

The catheter samples were harvested aseptically and prospectively collected and cultured at the end of ECMO support. After removal, the sections (5 cm) were cut from the intravascular region, split longitudinally, and immediately transported to the laboratory.

### Oxygenator culture

The oxygenator membranes were also harvested aseptically and prospectively collected and cultured. The membrane oxygenator was rinsed through the tube system with sterile physiological saline solution (0.9% w/v NaCl, twice) to remove blood. Subsequently, it was opened under sterile conditions. A hollow fiber membrane smear was taken from the opened membrane oxygenator, using a sterile swab.

### Microbiological examination

The catheter segments and oxygenator swab were submitted to the local microbiology laboratories for routine culture and antibiogram. Blood culture samples from patients at the time of catheter removal underwent standard culture methods. Bacterial growth from oxygenator swab cultures was assessed using a semi-quantitative method [15].

### Statistical analysis

All statistical analyses were performed using R software, version 3.6.2 (http://www.R-project.org) or SPSS version 25 (IBM corp., Armonk, NY). Continuous variables were described as means ± standard deviation and were compared between groups using the *t* test. Categorical variables were described as numbers (%) and were compared using the *χ*^2^ test. Two-tailed *p* values < 0.05 were considered to reflect statistical significance. To determine whether there was a difference in BSI and colonization, we calculated the incidence difference (per 1000 ECMO catheter days) between 2 groups and reported the associated 95% confidence interval (CI). Univariate logistic regression analysis was performed to evaluate the risk factors of BSI during ECMO. Significant factors (*p* < 0.05) were included in a multiple logistic regression analysis to determine the risk factors of BSI during ECMO. The backward stepwise (likelihood ratio) method was used for multivariate analysis, with entry and removal *p* values set at 0.05. We graphically compared occurrences of BSI between 2 groups using the Kaplan-Meier method and log-rank test. Also, the cox regression analysis was performed to evaluate the predictors of overall mortality.

## Results

### Patient characteristics

A total of 1740 ECMO catheter days in 192 patients were studied. The mean age was 58 ± 14.4 years and the male ratio was 64.1%. The intervention and control group each had 96 eligible patients enrolled during the study period. There was no significant difference of baseline characteristics between the two groups, except Charlson comorbidity index (2 vs. 2.5, *p* = 0.043) and ratio of RRT during ECMO (53.1% vs. 34.4%, *p* = 0.009, Table 1).

**Table 1:** Baseline characteristics of patients

Variables	Control (***n*** = 96)	Intervention (***n*** = 96)	***p***
**Male**	56 (58.3)	67 (69.8)	0.098
**Age**	56.7 ± 13.4	59.4 ± 15.2	0.182
**BMI**	22.6 ± 4.3	23.3 ± 4.5	0.323
**CCI**	2.0 ± 1.6	2.5 ± 1.7	0.043
**Diabetes**	19 (19.8)	20 (20.8)	0.858
**Immunocompromised**	11 (11.5)	4 (4.2)	0.060
**SOFA**	11.3 ± 3.7	10.3 ± 3.7	0.059
**APACHE II**	20.2 ± 7.5	19.3 ± 6.6	0.377
**Vasopressor**	82 (85.4)	76 (79.2)	0.257
**Site of care**			0.978
**Medical ICU**	17 (17.7)	18 (18.8)	
**Surgical ICU**	46 (47.9)	46 (47.9)	
**Mixed ICU**	33 (34.4)	32 (33.3)	
**Mechanical ventilation**	90 (93.8)	90 (93.8)	1.000
**Type of ECMO**			0.426
**Cardiac**	36 (37.5)	29 (30.2)	
**Respiratory**	47 (49.0)	56 (58.3)	
**ECPR**	13 (13.5)	11 (11.5)	
**ECMO mode**			0.147
**VV**	48 (50.0)	58 (60.4)	
**VA**	48 (50.0)	38 (39.6)	
**Cannula configuration**			0.342
**FV-JV**	46 (47.9)	56 (58.3)	
**FV-FA**	48 (50.0)	38 (39.6)	
**FV-FV**	2 (2.1)	2 (2.1)	
**ECMO duration**	8.9 ± 9.2	9.2 ± 10.0	0.822
^a^**Long-term ECMO support**	17 (17.7)	16 (16.7)	0.848
**RRT use during ECMO**	51 (53.1)	33 (34.4)	0.009
**RRT duration during ECMO**	9.7 ± 12.5	6.0 ± 5.4	0.108
**Oxygenator function test**	9 (9.4)	17 (17.7)	0.092
**Oxygenator change**	10 (10.4)	14 (14.6)	0.383

Values are expressed as mean ± standard deviation or *n* (%)

*BMI* body mass index, *CCI* Charlson comorbidity index, *SOFA* Sequential Organ Failure Assessment, *APACHE II* Acute Physiology and Chronic Health Evaluation II, *ICU* intensive care units, *ECMO* extracorporeal membrane oxygenation, *ECPR* extracorporeal cardiopulmonary resuscitation, *BSI* blood stream infection, *VV* veno-venous, *VA* veno-arterial, *FV* femoral vein, *JV* jugular vein, *FA* femoral artery, *RRT* renal replacement therapy

^a^Long term was defined as more than 2 weeks

### Comparison of infection incidence by intervention

The intervention of disinfection with 2% CHG/IPA of exposed circuits and hub significantly decreased the incidence of BSI during ECMO from 11.7/1000 ECMO catheter days to 2.3/1000 ECMO catheter days (difference 9.4, 95% CI 1.5–17.3, *p* = 0.019) in the study period (Table 2). As well, the incidence of ECMO catheter colonization was significantly higher in the control group (10.5/1000 ECMO catheter days vs. 2.3/1000 ECMO catheter days (difference 8.3, 95% CI 0.7–15.8, *p* = 0.032). The positive rate of oxygenator culture was higher in the control group (14% vs. 1.1%, *p* = 0.002). The incidence of VAP was higher in the control group (25.7% vs. 10.2%, *p* = 0.016). Other central venous catheter-related BSI was higher in the control group (21.1% vs. 7.9%, *p* = 0.023).

**Table 2:** Comparison of infection incidence by intervention

	Control (***n*** = 96)		Intervention (***n*** = 96)		Difference (95% CI)	***p***
	Events	Rate^**a**^	Events	Rate^**a**^		
**ECMO BSI**	10	11.7	2	2.3	9.4 (1.5–17.3)	0.019
**ECMO catheter culture positive**	9	10.5	2	2.3	8.3 (0.7–15.8)	0.032
**Oxygenator culture positive**	12	14.0	1	1.1	12.9 (4.7–21.1)	0.002
**Other CRBSI**	18	21.1	7	7.9	13.1 (1.8–24.5)	0.023

*ECMO* extracorporeal membrane oxygenation, *BSI* blood stream infection, *CRBSI* catheter-related bloodstream infection

^a^Rates are expressed per 1000 ECMO days. There were 855 ECMO days in the control group and 885 ECMO days in the intervention group

### ECMO complications and clinical outcomes

There were no significant differences of hemorrhage and thrombosis between two groups (Table 3). Although there were no significant differences of weaning rates from ECMO, ventilator duration, ICU stay, and hospital stay, the ICU mortality was significantly higher in the control group (38.5% vs. 21.9%, *p* = 0.012). Also, hospital mortality was significantly higher in the control group (41.7% vs. 24%, *p* = 0.009) and sepsis-related death was significantly higher in the control group (17.7% vs. 6.3%, *p* = 0.014).

**Table 3:** ECMO complications and clinical outcomes

Variables	Control (***n*** = 96)	Intervention (***n*** = 96)	***p***
**ECMO complications**			
**Hemorrhage**	22 (22.9)	25 (26.0)	0.615
**Thrombosis**	3 (3.1)	7 (7.3)	0.194
**ECMO weaning rates**	81 (84.4)	84 (87.5)	0.533
**Ventilator duration**	18.4 ± 21.5	24.3 ± 29.0	0.114
**ICU length of stay**	23.4 ± 22.2	27.3 ± 27.8	0.281
**Hospital length of stay**	50.2 ± 48.3	58.8 ± 46.2	0.208
**ICU mortality**	37 (38.5)	21 (21.9)	0.012
**Hospital mortality**	40 (41.7)	23 (24)	0.009
**Sepsis**-**related death**	17 (17.7)	6 (6.3)	0.014

Values are expressed as mean ± standard deviation or *n* (%)

*ECMO* extracorporeal membrane oxygenation, *ICU* intensive care unit

### Risk factors for BSI during ECMO

**Fig. 2:**
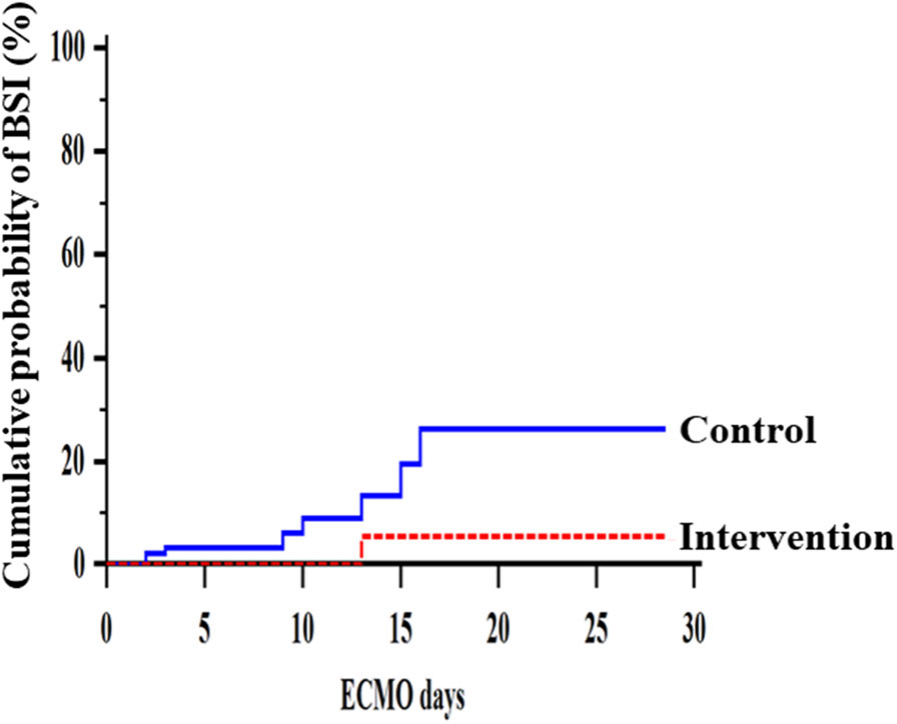
Occurrence of bloodstream infection by intervention. The intervention was significantly related to lower bloodstream infection rate (*χ*^2^ = 5.70, *p* = 0.017)

In the univariate regression analysis, long-term ECMO support of ≥ 2 weeks (odds ratio (OR) 3.88, 95% confidence interval (CI) 1.15–13.09, *p* = 0.029), 2% CHG/IPA intervention (OR 0.18, 95% CI 0.04–0.86, *p* = 0.031), RRT during ECMO (OR 4.20, 95% CI 1.10–16.04, *p* = 0.036), and oxygenator exchange (OR 4.00, 95% CI 1.10–14.49, *p* = 0.035) were significantly associated with BSI (Table 4). In the multivariate analysis, 2% CHG/IPA intervention (OR 0.16, 95% CI 0.03–0.76, *p* = 0.021) and oxygenator exchange (OR 5.13, 95% CI 1.32–20.00, *p* = 0.019) were significantly associated with BSI. The Kaplan-Meier curves showed that the intervention was significantly related to lower BSI rate (*χ*^2^ = 5.70, *p* = 0.017, Fig. 2).

**Table 4:** Risk factors of BSI during ECMO support

Variable	Univariate regression		Multivariate regression	
	OR (95% CI)	*p*	OR (95% CI)	*p*
^a^**Long-term ECMO support**	3.88 (1.15–13.09)	0.029		
**Intervention**	0.18 (0.04–0.86)	0.031	0.16 (0.03–0.76)	0.021
**RRT during ECMO**	4.20 (1.10–16.04)	0.036		
**Oxygenator exchange**	4.00 (1.10–14.49)	0.035	5.13 (1.32–20.00)	0.019

*OR* odds ratio, *CI* confidence interval, *ECMO* extracorporeal membrane oxygenation, *RRT* renal replacement therapy

^a^Long term was defined as more than 2 weeks

### Risk factor of overall mortality

In the univariate cox regression analysis, factors, which were significantly associated with overall mortality, included age ≥ 65 years (OR 1.74, 95% CI 1.05–2.88, *p* = 0.031), APACHE II (OR 1.04, 95% CI 1.01–1.08, *p* = 0.027), RRT during ECMO (OR 2.95, 95% CI 1.73–5.03, *p* < 0.001), and 2% CHG/IPA intervention (OR 0.49, 95% CI 0.29–0.82, *p* = 0.006). In the multivariate cox regression analysis, age ≥ 65 years (OR 1.98, 95% CI 1.20–3.28, *p* = 0.008), RRT during ECMO (OR 2.89, 95% CI 1.69–4.96, *p* < 0.001), and 2% CHG/IPA intervention (OR 0.52, 95% CI 0.31–0.87, *p* = 0.013) were significantly associated with overall mortality.

### Causative microorganisms of ECMO catheter colonization

The causative microorganisms cultured in 11 patients with ECMO catheter colonization are shown in Additional file 2. The main microorganisms identified in the control group were gram-positive bacteria such as *S*. *epidermidis* (3.1%), *Enterococcus faecalis* (1.0%), and other staphylococcus species (1.0%). *Acinetobacter baumannii* (3.1%) and Candida species (2.1%) were also found. One patient had both *Enterococcus faecalis* and *Candida tropicalis* infection. However, only Candida species, i.e., *Candida glabrata* (1.0%) and *Candida albicans* (1.0%), were found in the intervention group.

## Discussion

In this study, the incidence of BSI and ECMO catheter colonization was significantly lower in the intervention group. Furthermore, overall mortality and sepsis-related mortality was significantly lower in the intervention group. This inexpensive and readily available intervention, disinfection with 2% CHG/IPA of all exposed circuits and hubs, has the potential to reduce the BSI rate of patients on ECMO.

To our knowledge, there is no proven and effective intervention to prevent BSI during ECMO. For whatever reason, prophylactic antibiotic therapy in ECMO management failed to reduce the infectious complications [16, 17]. Currently, there are no studies specifically reporting the effects of preventive practices in ECMO patients, including antibiotic prophylaxis protocols.

Previously, we found a strong correlation between BSI and ECMO catheter colonization [1]. The microorganisms colonizing ECMO catheters were predominantly gram-positive cocci and Candida species. In a follow-up study, we confirmed the surfaces of ECMO catheters can become colonized with microorganisms that form a biofilm by using electron micrographs [18]. Microorganisms of the skin gain access at the ECMO catheter insertion wound and can migrate along the catheter’s subcutaneous tract into the intravascular catheters [19]. In particular, ECMO circuits and hub are more exposed to the skin and the environment than other intravascular catheters, and these exposed areas can be a potential source of catheter-related infections by the manipulation of hub or unintentional contact of cannula. Therefore, in addition to strict hand hygiene and aseptic techniques during insertion, proper ECMO circuit and hub care may be beneficial to the prevention of catheter associated infections. In this context, we have introduced chlorhexidine bathing of the entire exposed circuits and hub of ECMO in addition to the dressing of the catheter insertion site.

The findings of this study are supported by robust evidence that have demonstrated the safety and potential benefit of chlorhexidine bathing in critically ill patients [20–22]. In this study, the incidence of BSI in the control group was 11.7 per 1000 ECMO catheter days, which was similar to previous reports [4]. Our intervention significantly decreased the incidence of BSI. The effects of disinfection with CHG are supported by the difference in the catheter colonization in the intervention group as compared with the control group; gram-positive pathogens and *A*. *baumannii* were not found in the intervention group, only Candida species were found. Basically, CHG is more effective in gram-positive pathogen control as compared to gram-negative or fungal growth, because gram-negative BSI often originates from the lungs or digestive tract, not from the skin [20, 23–25]. However, *A*. *baumannii* can also be found on human skin or intravascular catheters and can often be detected from hospital environments [26]. A recent meta-analysis showed that CHG bathing significantly reduces colonization of *A*. *baumannii* and catheter-related infection in the ICU setting [21]. Nevertheless, it is still unclear whether this effect resulted from disinfection with CHG and/ or the mechanical cleansing effect. Therefore, further study is warranted.

In this study, 4 out of the 12 patients who had BSI during ECMO died of sepsis. The sepsis-related death was significantly lower in the intervention group. Especially, in the control group, the microorganisms for BSI and sepsis were the same (Additional file 3). We cannot show the causal correlation between BSI during ECMO and sepsis-related death. However, this microbiologic data could support the association between BSI and sepsis-related death in ECMO.

This study has several limitations including its nature as an uncontrolled trial and its small sample size. Both groups were not fully balanced in confounders such as RRT use and duration, proportion of immunosuppression, SOFA score, and oxygen function test. As well, the incidence of VAP of the control group was higher, which could impact on mortality. Generally, uncontrolled trials have a risk of over-estimating the treatment benefit as compared with randomized controlled trials. However, the intensity of the intervention was low, and the performance bias was minimized [27]. The intervention was only an extension of the area of disinfection with CHG. In addition, to reduce history bias, we compared the rates of BSI and mortality in ECMO patients before and after this study period (additional file 4). As stopping the intervention, the rates of BSI and mortality has increased in the following period after the study.

Currently, there are no standardized guidelines for the prevention of infection during ECMO support. It is evident that acquired infection during ECMO leads to worse clinical outcomes [28]. Notwithstanding, individual infection prevention policies are adopted according to institutional preference [29, 30]. Most of the represented strategies used CHG disinfection only for the cannula insertion sites [31]. Despite the critical importance of infection precaution in patients on ECMO, evidence is still lacking. In particular, ECMO is a highly resource-demanding procedure, which requires a significantly high cost [32, 33]. Given that ECMO is already quite expensive and resource-intensive, this intervention could be an inexpensive and cost-effective strategy that can lead to enormous cost savings. In this context, this study is meaningful in that it represents the first data on the effective prevention of infection in ECMO cases. This study would be beneficial in creating an evidence-based guideline for acquired infection control during ECMO. However, a large-scale, multi-center, randomized controlled study is required to confirm the results of this study.

## Conclusions

In this uncontrolled before-and-after study, the chlorhexidine bathing of exposed circuits and hub was significantly associated with lower incidence of BSI during ECMO. As well, the incidence of ECMO catheter colonization was lower, especially in gram-positive pathogens. The intervention group showed lower overall and sepsis-related mortality without any adverse effect after the intervention. This intervention could be a simple, effective strategy to decrease the BSI on ECMO.

## Supplementary information

**Additional file 1.** Study protocol.

**Additional file 2.** The culture results of ECMO catheters.

**Additional file 3.** The microorganisms for bloodstream infection among the sepsis related mortality.

**Additional file 4.** The rates of mortality and bloodstream infection in extracorporeal membrane oxygenation patients before and after this study period.

## Data Availability

The datasets used and/or analyzed during the current study are available from the corresponding author on reasonable request.

## Abbreviations

ECMO: Extracorporeal membrane oxygenation
BSI: Blood stream infection
RRT: Renal replacement therapy
CHG: Chlorhexidine gluconate
IPA: Isopropyl alcohol
CFU: Colony forming units
IDSA: Infectious Disease Society of America
VAP: Ventilator-associated pneumonia
OR: Odds ratio
CI: Confidence interval
