# Supplementary material for: Chlorhexidine bathing of the exposed circuits in extracorporeal membrane oxygenation: an uncontrolled before-and-after study
Source: Crit Care. 2020 Oct 6;24:595. doi: 10.1186/s13054-020-03310-w (PMC7538059; doi:10.1186/s13054-020-03310-w)
Supplement: Supplementary file 1 — Additional file 1. Study protocol. [file 13054_2020_3310_MOESM1_ESM.zip › additionalfile1.docx]

**Additional file 1. study protocol**

This an uncontrolled before-and-after, retrospective and prospective, single center study. The participating site was a tertiary care hospital, which conduct ECMO and transplantation for cardiorespiratory failure. We enrolled patients aged at least 18 years who required ECMO. We excluded patients who were terminated ECMO within 48 hours or had a chlorhexidine allergy. The intervention was a daily disinfection of all exposed circuits and hub including ECMO catheter insertion sites with 2% CHG/IPA during ECMO support. In order to minimize the performance bias, we trained the intervention practice for one month in August 2018. The intervention group was prospectively enrolled after obtaining consent from the patient or guardian from September 2018 to August 2019 and collected data. The control group was retrospectively collected data on patients who performed ECMO before the intervention from March 2017 to July 2018. During the pre-intervention period, standard practice was for all ECMO patients to receive daily 2% CHG disinfection at the ECMO catheter insertion site according to the infection precaution policy of the hospital. Except for the intervention, all patient care and ECMO management were performed identically. Basically, all patients with central lines received daily 2% CHG bathing to clean neck and chest. This project was approved by the Pusan National University Yangsan Hospital Institutional Review Board (05-2018-149) in September 2018 and the study was registered on the Clinical Research Information Service (KCT 0004431). In the intervention group, written informed consent for enrollment or consent to continue and use patient data was obtained from each patient or their legal surrogate. The control group was waived from consent due to retrospective data collection.
