# Supplementary figures and images for: Chlorhexidine bathing of the exposed circuits in extracorporeal membrane oxygenation: an uncontrolled before-and-after study
Source: Crit Care. 2020 Oct 6;24:595. doi: 10.1186/s13054-020-03310-w (PMC7538059; doi:10.1186/s13054-020-03310-w)

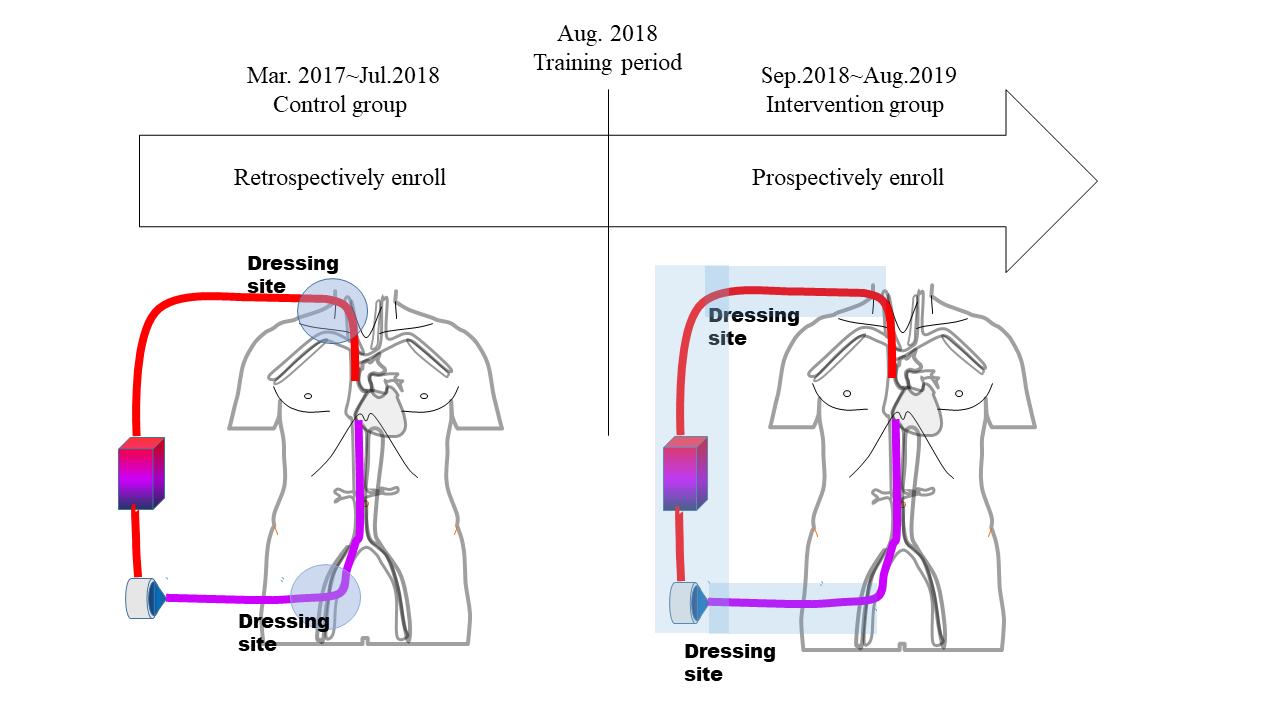

Supplement: Supplementary file 1 — Additional file 1. Study protocol. [file 13054_2020_3310_MOESM1_ESM.zip › additional file 1.tif]
