# Supplementary material for: Chlorhexidine bathing of the exposed circuits in extracorporeal membrane oxygenation: an uncontrolled before-and-after study
Source: Crit Care. 2020 Oct 6;24:595. doi: 10.1186/s13054-020-03310-w (PMC7538059; doi:10.1186/s13054-020-03310-w)
Supplement: Supplementary file 2 — Additional file 2. The culture results of ECMO catheters. [file 13054_2020_3310_MOESM2_ESM.docx]

**Additional file 2. The culture results of ECMO catheters**

| **Variables** | **control (n=9)** | **intervention (n=2)** |
| --- | --- | --- |
| **Microbiology of ECMO catheter** |  |  |
| **Staphylococcus epidermidis** | 3 |  |
| **Enterococcus faecalis** | 1* |  |
| **Gram positive, other** | 1 |  |
| **Acinetobacter baumanii** | 3 |  |
| **Candida tropicalis** | 2* |  |
| **Candida glabrata** |  | 1 |
| **Candida albicans** |  | 1 |

Values are expressed as n.

One patient had both *Enterococcus faecalis* and *Candida tropicalis* infection (*).
