# Supplementary material for: Chlorhexidine bathing of the exposed circuits in extracorporeal membrane oxygenation: an uncontrolled before-and-after study
Source: Crit Care. 2020 Oct 6;24:595. doi: 10.1186/s13054-020-03310-w (PMC7538059; doi:10.1186/s13054-020-03310-w)
Supplement: Supplementary file 3 — Additional file 3. The microorganisms for bloodstream infection among the sepsis related mortality. [file 13054_2020_3310_MOESM3_ESM.docx]

**Additional file 3. The microorganisms for bloodstream infection among the sepsis related mortality**

| Group | Microorganism for BSI | Microorganism for sepsis |
| --- | --- | --- |
| Control | Pseudomonas aeruginosa | Pseudomonas aeruginosa |
| Control | Enterococcus faecium | Enterococcus faecium |
| Control | Acinetobacter baumanii | Acinetobacter baumanii |
| Intervention | Staphylococcus epidermidis | Pseudomonas aeruginosa |

BSI; bloodstream infection.
