# Supplementary material for: Chlorhexidine bathing of the exposed circuits in extracorporeal membrane oxygenation: an uncontrolled before-and-after study
Source: Crit Care. 2020 Oct 6;24:595. doi: 10.1186/s13054-020-03310-w (PMC7538059; doi:10.1186/s13054-020-03310-w)
Supplement: Supplementary file 4 — Additional file 4. The rates of mortality and bloodstream infection in extracorporeal membrane oxygenation patients before and after this study period. [file 13054_2020_3310_MOESM4_ESM.docx]

**Additional file 4. The rates of mortality and bloodstream infection in extracorporeal membrane oxygenation patients before and after this study period**

|  | Before the study  (n=94) | Control (n=96) | Intervention (n=96) | After the study (n=83) |
| --- | --- | --- | --- | --- |
| Duration | Jan 2016 ~Feb 2017 | Mar 2017~Jul 2018 | Sep 2018~Aug 2019 | Sep 2019~Aug 2020 |
| Total ECMO days | 1,075 | 855 | 885 | 828 |
| BSI events | 16 | 10 | 2 | 13 |
| Rate^a^ | 14.9 | 11.7 | 2.3 | 15.7 |
| Mortality | 41 (43.6) | 40 (41.7) | 23 (24) | 28 (33.7) |

^a^ Rates are expressed per 1000 ECMO-days.

ECMO; extracorporeal membrane oxygenation, BSI; bloodstream infection.
